# Protein Array Patterning by Diffusive Gel Stamping

**DOI:** 10.1371/journal.pone.0046382

**Published:** 2012-10-10

**Authors:** Mekhail Anwar, Piyush B. Gupta, Raja Palaniapan, Paul Matsudaira

**Affiliations:** 1 Whitehead Institute for Biomedical Research, Cambridge, Massachusetts, United States of America; 2 Whitehead-Massachusetts Institutes of Techonology Bioimaging Center, Cambridge, Massachusetts, United States of America; 3 Department of Electrical Engineering and Computer Science, Massachusetts Institute of Technology, Cambridge, Massachusetts, United States of America; 4 Department of Biology, Massachusetts Institute of Technology, Cambridge, Massachusetts, United States of America; 5 Department of Biological Engineering, Massachusetts Institute of Technology, Cambridge, Massachusetts, United States of America; Aligarh Muslim University, India

## Abstract

Proteins and small molecules are the effectors of physiological action in biological systems and comprehensive methods are needed to analyze their modifications, expression levels and interactions. Systems-scale characterization of the proteome requires thousands of components in high-complexity samples to be isolated and simultaneously probed. While protein microarrays offer a promising approach to probe systems-scale changes in a high-throughput format, they are limited by the need to individually synthesize tens of thousands of proteins. We present an alternative technique, which we call diffusive gel (DiG) stamping, for patterning a microarray using a cellular lysate enabling rapid visualization of dynamic changes in the proteome as well protein interactions. A major advantage of the method described is that it requires no specialized equipment or *in-vitro* protein synthesis, making it widely accessible to researchers. The method can be integrated with mass spectrometry, allowing for the discovery of novel protein interactions. Here, we describe and characterize the sensitivity and physical features of DiG-Stamping. We demonstrate the biologic utility of DiG-Stamping by (1) identifying the binding partners of a target protein within a cellular lysate and by (2) visualizing the dynamics of proteins with multiple post-translational modifications.

## Introduction

Systems-scale analysis of the proteome, for example the identification of binding partners or post-translational modifications, requires thousands of components in high-complexity samples to be isolated and simultaneously probed. Protein patterning on microarrays has been shown to be useful for high-throughput analyses, probing with multiple targets, and for minimizing reagent quantities [1]–[3]. Additionally, by simultaneously analyzing multiple samples [4], [5], microarrays enable rapid visualization of differential changes. The equipment needed for fabricating microarrays can restrict their use, although novel approaches such as patterning with an agarose-based stamp have been implemented [6]. Notwithstanding these advantages, the manufacture and use of protein microarrays has been significantly hindered by the need to synthesize thousands of partial or full-length proteins. In one approach, the yeast proteome was expressed from individual clones, purified, and then arrayed [7]. Cellular lysates are often the most appropriate choice for protein studies because they retain all of the cellular components with the appropriate post-translational modifications. Alternative methods have attempted to simplify protein synthesis by translating thousands of individual proteins directly on the microarray surface using *in vitro* translation [8], [9]. Others have patterned fractionated cellular lystates to generate arrays [10], [11], requiring an HPLC and microarray spotter. The method presented here addresses these limitations by patterning an array using a cellular lysate without the need for any specialized lab equipment, enabling rapid visualization of the proteome and protein interactions.

The precursors to protein microarrays are membrane blots transferred from cellular lysates separated by polyacrylamide gel electrophoresis (SDS-PAGE). Screening of novel binding interactions can be accomplished through the use of Far-western blots [12], but it is often difficult to identify the binding partners as they are membrane-bound. Thus, while gel-based blotting methods make use of cellular lysate as a binding substrate, a major limitation is that the majority of protein is lost to analysis on the membrane. Western blots [13] bypass this issue by probing with antibodies with known targets, but this significantly restricts throughput and limits the discovery of novel interactions. We overcome this by integrating the patterning of a cellular lysate on a microarray substrate with focused, biochemical studies, such as mass spectrometry analysis, by retaining the majority of the protein sample within the gel, making it available for further analysis.

While protein microarrays can characterize samples based on their specific binding to patterned proteins, mass spectrometry identifies proteins *de novo* via sequencing, enabling novel protein binding partners to be discovered. However, mass spectrometry, which is limited in throughput, must be combined with other techniques in order to determine binding interactions. Often, the proteins of interest for a particular study are those that change in response to specific biologic stimuli or genetic manipulations. In principle, restricting microarray analyses to such proteins would significantly reduce sample complexity in a hypothesis-relevant manner and would allow for a synergistic combination with mass spectrometry.

**Figure 1:**
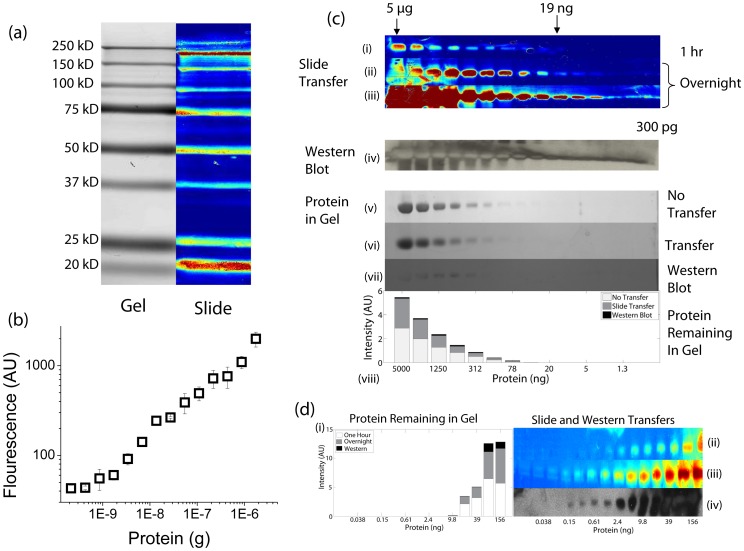
Characterizing Western Transfer Efficiency. (a) Transfer of a protein ladder. A BioRad Blue protein ladder was run on a 10% SDS acrylamide gel and diffusively transferred for one hour to an aminosilane slide. The slide was scanned with the Axon 4000B microarray scanner, and the gel was scanned with the Typhoon fluorescent scanner after transfer. There is a direct spatial correspondence between the protein in the gel and the slide. All molecular weights transfer. It should be noted, that the lower molecular weight proteins diffuse more readily than the higher molecular weight proteins. Therefore, the lower molecular weight proteins will transfer faster, but also diffuse laterally, creating a wider band. As can be seen from the gel scan, the vast majority of the protein remains in the gel. (b) Detection limit of 30 minute transfer with DiG-Stamping. The minimum detection limit was 10 ng of biotin BSA after transferring for 30 minutes. The average of 3 gels with standard deviation is shown. (c) Comparison of Western blot with DiG-Stamping. Serial dilutions of biotin BSA were run on identical SDS gels (10%) and either diffusively transferred for 1 hour (i) or overnight [ii (low gain) and iii (high gain)] to an aminosilane slide, or transferred via Western blot to nitrocellulose (iv). Slides and Western Blot were processed as described in Protocol S1. After 1 hour, 19 ng (i) could be detected on the diffusive blot, and after overnight transfer, 300 pg (iii) could be detected. 300 pg was readily detected using the Western blot (iv). To establish the amount of protein that remains for analysis in the gel after transfer, a gel without transfer was stained with Commassie blue, and scanned (v), providing a reference. After overnight transfer to a slide (vi), almost all of the protein remains in the gel. In the case of the Western blot (vii), very little of the protein remains in the gel after transfer. The quantification of the amount of protein remaining in each gel is shown. (d) Detection limit comparison of Western blotting to DiG-Stamping. To further probe the detection limits, the same experiment as in (b) was repeated, but with smaller amounts of protein. Western blot showed a limited of ∼150 pg, and the overnight transfer showed a limit of ∼19 pg.

Towards this objective, we have developed a novel method, DiG-Stamping, that allows for biologically-motivated complexity reduction in high-content samples and subsequent unbiased discovery of novel protein interactions and proteome dynamics. The basics of DiG-Stamping are straightforward. The protein array is assembled by transferring a cellular lysate (or subset thereof) separated via SDS-PAGE to a chemically functionalized slide by direct contact with the gel (Fig. 1a). The resulting replica of a patterned cellular lysate enables integration of high sensitivity, low-reagent quantity, multi-channel microarray techniques with the simplicity of a Far-Western blot, while simultaneously preserving the vast majority of the protein sample for de-novo analysis with mass spectrometry. This enables rapid visualization of the proteome and protein interactions, allowing study of dynamic changes as well as comparisons of the proteome across cell lines and physiologic conditions, while enabling focused biochemical studies to be performed on the regions of interest.

## Materials and Methods

### Characterization of Transfer

To characterize the transfer efficiency, serial dilutions of Alexa 555 Ovalbumin ranging from 0.3 µg to 19 pg, were run on a 1 mm, 15 well 10% Tris glycine gel, under denaturing conditions (Tris-Glycine-SDS Buffer, pH) in triplicate. The gels were transferred for 60 minutes to aminosilane-coated glass slides with no applied voltage according to the protocol in Protocol S1. A wide variety of protein and DNA binding substrates compatible with microarray applications exist. These are largely distinguished by their surface-binding characteristics, and include slides coated with a 3D matrix (e.g. hydrogel, nitrocellulose), slides with a charged surface, and slides functionalized for covalent binding [14]. Aminosilane slides – which bind proteins via electrostatic interactions – were selected for their low autofluorescence, chemical stability, and effectiveness in binding a wide variety of proteins.

The slides were scanned with the Axon 4000B (Gain = 600) (Fig. S1 1e), and the amount of protein transferred was quantified by summing the total fluorescence for each band in MATLAB. The values from the three slides were averaged, and the mean and standard deviation were plotted. To establish the effect of the electric field on transfer, two sets of gels (three gels each), were transferred, with one set subjected to 50 V applied to conducting plates placed above and below the gel and slide, 0.5 cm apart, and one set of gels with no applied voltage. To look at the effect of time on the transfer, gels were transferred as described above, for 10, 60, 120 and 1000 minutes. To establish the effect of gel thickness, 0.5 mm, 0.75 mm, 1.0 mm and 1.5 mm gels were made using the BioRad mini-gel system, with a 10% Tris-Glycine denaturing gel. The gels were run in triplicate, transferred and quantified as described above.

### Western Blot

To investigate the limit of detection for a brief (30 minute) gel transfer assay, we measured the detection efficiency by using biotinylated BSA (bovine serum albumin), probed with streptavidin Cy3. 10% Tris-Glycine, 15 well, 1 mm gels were run in triplicate with serial dilutions of biotinylated BSA ranging from 10 µg to 100 pg and transferred for 1 hour. The slide was probed with 20 nM solution of streptavidin Cy3, imaged and quantified (Protocol S1).

To compare the DiG-Stamping method to a standard Western blot (Protocol S1), gels were run with serial dilutions of biotinylated BSA from 5 µg to 300 pg, and transferred to aminosilane slides for 1 hour and overnight, and probed with Streptavidin Cy3 as described above. The overnight slide was scanned at normal gain (600), and also at high gain (900). Gel images were obtained by staining with Coomassie-based Simply Blue, and scanned. The intensity of each band was quantified by taking the total intensity and subtracting the background with MATLAB. To further probe detection sensitivity, the experiment was repeated with smaller amounts of biotinylated BSA, ranging from 156 ng to 19 pg.

### Identification of Protein-Protein Interactions

We describe the preparation of HeLa cell lysate in Protocol S1. 25 µl of cell lysate was added to 100 ng of biotinylated BSA (bBSA). 3 µl of 10× sample buffer was added to the mixture, and the solution was heated at 95°C for 3 minutes. The sample was run (lane 1) on a 10% SDS mini-gel. In the neighboring lane (lane 2) a Cy5 fluorescently-labeled ladder is run. The gel was transferred for 1 hour, probed with a 1∶1000 dilution of Cy3 streptavidin for one hour, and imaged (Protocol S1). The raw slide images were processed, and the fluorescence intensity was averaged and plotted as a function of position, allowing for quantitative peak identification. A spatial mapping of the gel to the slide was obtained by matching the positions of the ladder bands on the gel with the bands on the slide and the corresponding position of the Cy3 band on the slide was excised from the gel. Proteins were extracted and analyzed on a the QSTAR mass spectrometer [15]. To further isolate the protein of interest, one half of the band was eluted in 100 µl of PBS at 4 degrees for 48 hours, and immunoprecipitated with 20 µl of streptavidin magnetic beads per the Dynabeads protocol. The elutant was directly run on the mass spectrometer.

### T-47D – Heregulin Protein Assay

Preparation of T-47D cells lysates with and without Heregulin-β treatment are described in Protocol S1. Briefly, cell lysates from cells with and without Heregulin-β treatment were immunoprecipitated with anti-phosphotyrosine beads, and were run on a 10% SDS-PAGE gel. The gel was transferred according to Protocol S1. The slides were blocked overnight in 5% BSA in PBS, and then incubated with 1∶100 dilutions of anti-phosphotyrosine 635 antibody and anti-ubiquitin 488 antibody in 5% BSA in PBS overnight. The slides were washed in PBS, spun dry, and scanned with the Axon 4000A.

## Results

To identify the parameters that modulate protein transfer and detection with DiG-Stamping, we characterized the transfer of a serial dilution of fluorescently-labeled Ovalbumin with respect to concentration, gel thickness, applied electric field (Fig. S1 1a–c), and molecular weight (Fig. 1a). Quantification of the fluorescence signal on the slide showed that the amount of protein transferred was unaffected by an applied electric field, but linearly dependent on protein concentration and gel thickness. We observed, moreover, that the patterned protein band is in a Gaussian distribution and that the quantity of protein transferred as a function of time fit well to a first-order exponential equation (Fig. S2 1d), further indicating that a diffusion-mediated motive force is responsible for protein transfer. This is consistent with the fact that there is no electric field within a conductive material bounded by insulating layers, in this case a conductive hydrogel surrounded by a glass slide and air. This results from a redistribution of charge near the surface to cancel out the applied external electric field, leaving diffusion as the sole motive force (Fig. S2). Since diffusion is the motive force for transfer, this technique is applicable to any gel type, including native and 2-D gels.

In order to demonstrate the applicability of this technique to biological assays and compare it to conventional methods, we used DiG-Stamping to identify protein-protein interactions in comparison with Western blotting (Fig. 1b–d). To gauge detection efficiency, we transferred serial dilutions of biotin-BSA to glass slides and probed them with streptavidin Cy3. Quantification of the bands showed that there is a 10 ng detection limit after 30 minutes (Fig. 1b). To directly compare the detection limits by DiG-Stamping with Western blotting (Fig. 1c–d), we transferred biotin-BSA to a slide via DiG-Stamping and to nitrocellulose via electrophoresis. At short transfer times (e.g. 1 hr), 300 pg (Fig. 1c iii) can be visualized following Western blotting, whereas the detection limit following DiG-Stamping is approximately 10–20 ng (Fig. 1c ii). At longer transfer times (e.g. overnight), 20 pg (Fig. 1d ii) can be visualized on the slide following DiG-Stamping, compared with 150 pg (Fig. 1 d iv) following Western blotting. Again, while very little protein remains in the gel following Western blotting, virtually all (>90%) of the protein remains in the gel after the DiG-Stamping procedure. Thus, although longer transfer times are required in high-sensitivity applications with DiG-Stamping, it is possible to detect smaller amounts of protein compared with Western blotting, while preserving protein for further analysis.

**Figure 2:**
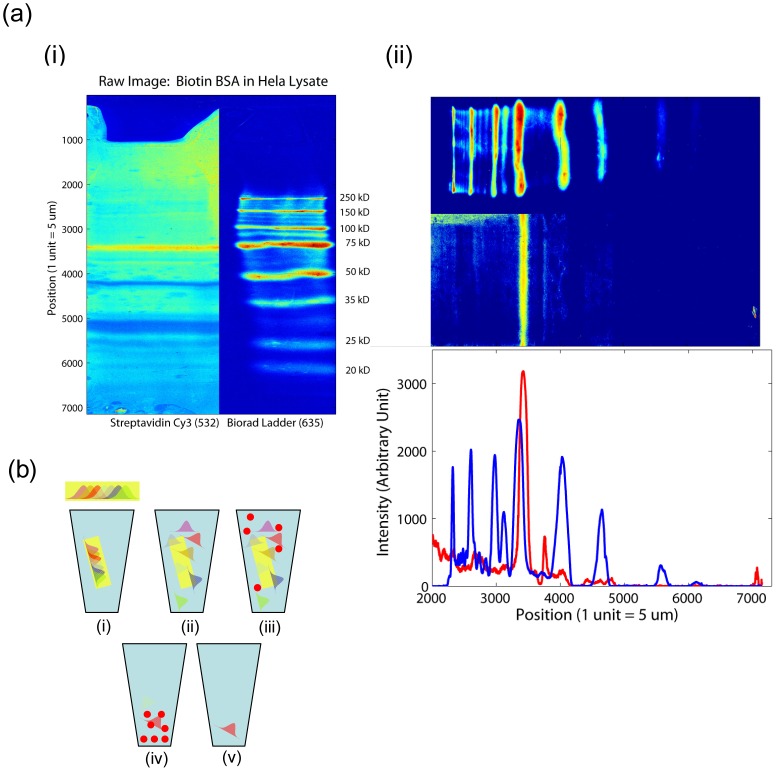
Identifying Protein-Protein Interactions. (a.i) HeLa cell lysate with Biotin BSA probed with streptavidin Cy3. The Cy3 channel is shown on the left, and the Cy5 channel showing the ladder in the neighboring lane, is on the right. The images shown are the unprocessed images. The lower molecular weight bands have a wider width. (a.ii) The images are filtered using a 2D Weiner filter in MATLAB, rotated 90 degrees, and thresholded to more clearly delineate the streptavidin band. This is useful for weaker signals which must be distinguished from the background, as well as to more accurately define the peaks of each band, enabling more accurate mapping to the gel. The filtered image is then averaged across the gel lane, further increasing signal to noise, and the signal as a function of position is plotted. The Cy3 trace (streptavidin) is plotted in red, and the ladder is plotted in blue. The position of the ladder peaks allows alignment of the gel and the transfer slide. (b) To further isolate the protein that is in the excised band, ½ of the band is eluted in PBS (i) over 48 hours (ii). Streptavidin coated magnetic beads are placed in solution (iii), allowed to bind, and then washed (iv). The beads are eluted in 8 M guanidine HCL (v), and the resulting solution is subjected to mass spectrometry.

We next investigated whether DiG-Stamping enables the probing of protein interactions in a format that allows for the identification of residual protein remaining in the gel. In principle, this could be accomplished by identifying the location of a binding event on the slide and then extracting the corresponding location in the gel. To demonstrate the feasibility of this approach, we used the biotin-streptavidin system to detect the binding partner(s) of streptavidin within a high-complexity cellular lysate. To do so, we added biotinylated-BSA (bBSA) and a fluorescent-labeled molecular protein ladder to cellular lysate from the HeLa cell line, transferred the lysate mixture following SDS-PAGE to a glass slide by DiG-Stamping, and then probed with streptavidin Cy3 (Fig. 2a). The gel position corresponding to the Cy3 band on the slide is excised and analyzed via mass spectrometry

This resulted in the identification of Albumin (ALB Protein), as well as several other proteins (Data S1 Table 1) in the excised band. Reasonable assumptions (e.g. known subcellular localization) regarding binding partners could eliminate several candidate binding proteins. Additionally, further spatial resolution can be obtained with the use of gradient gels or longer gels. To rigorously confirm that the candidate target protein (bBSA) bound the probe (streptavidin), we eluted the protein from the remaining one-half gel band, and immunoprecipitated albumin (Fig. 2b). This resulted (Data S1 Table 2) in the unique identification of the albumin as the target protein. This proof-of-principle experiment demonstrates that, since the vast majority of protein remains in the gel, DiG-Stamping enables mass spectrometry techniques to be synergistically combined with microarray technology.

Although the proteome contains tens of thousands of proteins, often only a subset of these are of interest and warrant further study in any given study; these could include proteins that interact with a target, change in expression level, or post-translational modification in response to a particular stimulus. In principle, DiG-Stamping could allow for biologically relevant complexity-reduction in lysates by first identifying dynamic protein changes on patterned microarrays, prior to selective follow-up analysis by mass spectrometry. This approach could help reduce the low-throughput bottleneck of mass spectrometry. Additionally, quantitative mass spectrometry (iCAT [16] and iTRAQ [17] for *in-vitro* labeling, and SILAC [18], [19] for *in-vivo* labeling) can be integrated with DiG-Stamping by labeling the lysate prior to patterning.

To examine the feasibility of this, we analyzed the changes in the immunoprecipitated phosphoproteome of breast cancer (T-47D) cells upon activation of the Her2/Neu receptor with Heregulin-β [20] as a function of time. Treatment of T-47D breast cancer cells with Heregulin-β induces cell migration after 3 hours of stimulation. The nature of the intracellular switch associated with the acquisition of migratory behavior is poorly understood, but presumably involves alterations in the proteome of the T-47D cells. We decided to use DiG-Stamping to examine T-47D cell protein phosphotyrosine modification because of its biologic importance for intracellular signaling cascades. In addition, phosphotyrosinated proteins are the smallest fraction of the phosphoproteome [21], thus reducing complexity while still representing a significant portion of signaling activity [22].

**Figure 3:**
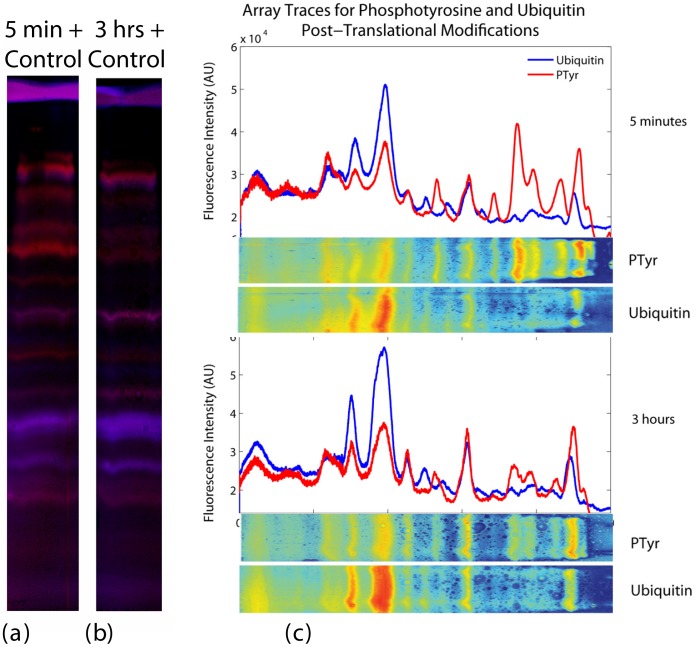
Identifying Protein-Protein Interactions. (a) Immunoprecipitates of phosphorylated proteins from T-47D (Manassas, VA) lines grown in media, treated with Heregulin for 5 minutes, and 3 hours, and control, in media without Heregulin, are mixed together, run on an SDS-PAGE gel and transferred overnight. The slides are probed with anti-ubiquitin (blue) and anti-phosphotyrosine (red). (b) The experiment is repeated, with cells treated with Heregulin for 3 hours. (c) The images from (a) and (b) are separated by color channel, thresholded, and filtered. (PTyr = anti-Phosphotyrosine, Ub = anti-Ubiquitin). The images are horizontally averaged across the gel lane, and plotted as a function of position. Clear differences between the phosphorylated states can be seen. The PTyr and Ub traces are markedly different, indicating that a distinct subset of phosphorylated proteins are ubiquitinylated. Since each lane is mixed with an equal quantity of control lysate, the differences shown here are solely due to the changes in phosphorylation from 5 minutes to 3 hours of treatment with Heregulin. Additionally, the ubiquitin traces can be seen to clearly overlap selected phosphorylation peaks, indicating proteins that have dual post-translational modifications.

The unprocessed slide images (Fig. 3a,b) show transfers of mixtures of control (no Heregulin-β treatment), with samples that have been stimulated with Heregulin-β for 5 minutes and 3 hours, respectively, probed with fluorescent anti-phosphotyrosine and anti-ubiquitin. The images are shown in Fig. 3c, with the traces of horizontally averaged images. Slides from each time point contain a 1∶1 mixture of the treated lysate and the control lysate. This gives each trace an offset equal to the control (no treatment), and the changes visualized represent the difference between the short term (5 minute) and long term (3 hour) phosphorylation states. In the case where cells are grown in SILAC labeled media, the relative concentrations of the control and treated proteins could then be determined via mass spectrometry.

Following this analysis, simultaneous visualization of both the phosphoproteome, as well as phosphoproteins with an ubiquitin post-translational modification, were directly compared across short-term and long-term Heregulin-β stimulation. Significant changes in the phosphoproteome could be readily identified, while levels of proteins having dual phosphorylation and ubiquitinylation modifications did not significantly change over time following Heregulin-β treatment (Fig. 3). This biological observation was not anticipated *a priori*, providing a demonstration that DiG-Stamping can provide novel information that may help direct further analyses.

## Discussion

Methods for the analysis of novel proteins and small molecule interactions are essential for the study of biological systems. We have developed and demonstrated a novel technique, DiG-Stamping, to rapidly fabricate protein microarrays by leveraging the use of cellular lysates. The primary advantage of the proposed technique is that it enables rapid visualization of binding interactions (with multiple ligands simultaneously) as well as proteome-level changes, with high sensitivity and minimal reagents. DiG-Stamping does not require any specialized laboratory equipment, making it accessible to all researchers. Furthermore, by utilizing only a small amount of protein for detection, DiG-Stamping enables further assay integration with existing biochemical techniques by preserving the majority of the protein sample in the gel.

Until now, the partial transfer of proteins from a gel to membrane-based substrate has been limited by the sensitivity of Western Blots. With the integration of recently developed microarray technology, as demonstrated here with DiG-Stamping, it is feasible to transfer only a portion of the protein sample without compromising protein detection sensitivity. As with all protein assays, the fundamental detection limits are set by the amount of protein sample being probed, combined with the sensitivity of the assay and detector itself. Therefore, the partial transfer of a quantity of protein by passive diffusion – as opposed to the complete transfer of the sample via electrophoresis– will inherently have a lower detection limit when the same method (e.g. Western Blotting) is used for detection. This was illustrated by Kurien et al [23], who demonstrated passive transfer of gel-separated proteins to nitrocellulose with a similar goal of preserving protein within the gel for further experimentation; however, their method allows for sample quantities down to only 1 µg, as opposed to the 20 pg demonstrated here. In a direct comparison of DiG-Stamping and traditional Western Blotting using identical gels, we showed that DiG-Stamping is more sensitive, despite the fact that only a fraction of the total sample was transferred to the slide. Although traditional protocols utilizing a primary and secondary antibody can be used with DiG-Stamping, one advantage of DiG-stamping is that primary antibodies can often be conjugated to small-molecule fluorophores without loss of binding specificity, thereby simplifying the assay protocol.

The primary limitation of DiG-Stamping in comparison to spotted arrays is the preferential transfer of high-abundance and low molecular-weight proteins (LMWPs), and the lateral diffusion of LMWPs diminishing spatial resolution. This makes isolation of individual proteins more difficult, but this is consistent with the limitations of all gel-based techniques. Care must also be taken in correlating the protein position on the slide image to the gel.

Gel transfer efficiency and protein detection limits were characterized using a fluorescently-tagged streptavidin as a secondary binding agent. This allowed for accurate comparison across both Western blots and DiG-Stamping, but the robust nature of the biotin-streptavidin bond does not reflect typical protein-protein interactions. To address this, and illustrate the applicability of the current technique to typical protein-protein interactions involving antibody identification of a target, we have also demonstrated simultaneous labeling of the T-47D lysate with fluorescently-tagged antibodies.

As illustrated by the analysis of T-47D cancer cell migration, the approach presented here allows for reduction in the complexity of a sample in a hypothesis-driven manner by identifying relevant dynamic changes in subsets of the proteome by DiG-Stamping. These relevant protein components can then, in principle, be identified by mass spectrometry, since the vast majority of protein following DiG-Stamping remains in the gel. This synergistic combination of easily fabricated protein microarrays and mass spectrometry addresses the low-throughput limitations of mass spectrometry.

As we have shown here, DiG-Stamping can be applied to screening for protein-protein interactions. In principle, any set of proteins can be patterned, and probed against multiple targets, using a fraction of the sample required in typical Western Blot applications. In the present study, these advantageous features of microarrays were used here to identify multiple post-translational modifications associated with cancer cell migration. Many other applications can be envisioned, e.g. patterning proteins associated with specific signaling pathways and subsequently probing either with other proteins or small-molecules.

## Supporting Information

Figure S1 **Characterizing Transfer Efficiency.** (a) Protein transfer versus concentration. (b) Protein transfer versus electric field. (c) Protein transfer versus gel thickness. (d) Protein transfer versus time. (e) A sample image of serial dilutions transferred to an aminosilane slide.
(TIF)

Figure S2 **Gel transfer mechanism.**
(TIF)

Protocol S1 **Reagents, Equipment and Procedure for Diffuse Gel Stamping; Hela Cell Lysate Preparation; T47D Culture and Lysate Preparation; Western Blot Methods.** Detailed figure captions.
(PDF)

Data S1 **Table 1: Proteins from Extracted Gel Band.** Table 2: Table 2: Proteins from immunoprecipitated Gel band.
(PDF)
