# Supplementary figures and images for: Protein Array Patterning by Diffusive Gel Stamping
Source: PLoS One. 2012 Oct 10;7(10):e46382. doi: 10.1371/journal.pone.0046382 (PMC3468578; doi:10.1371/journal.pone.0046382)

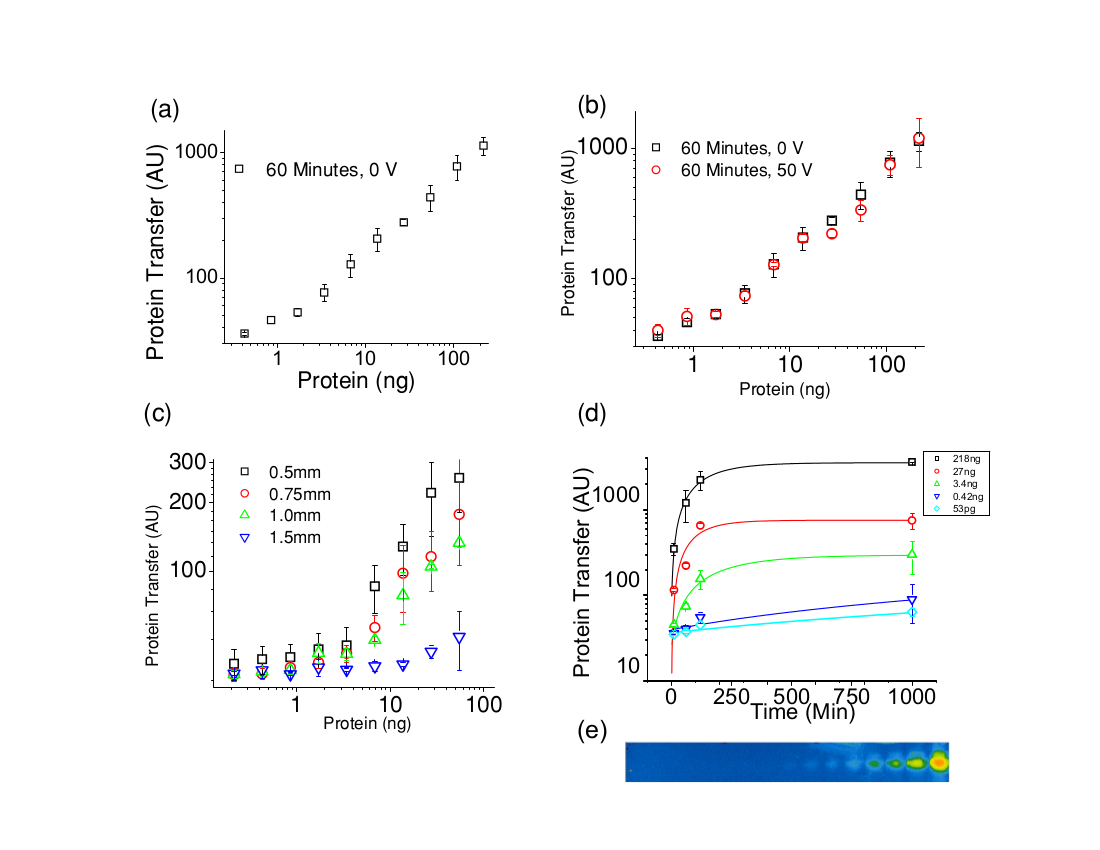

Supplement: Figure S1 — Characterizing Transfer Efficiency. (a) Protein transfer versus concentration. (b) Protein transfer versus electric field. (c) Protein transfer versus gel thickness. (d) Protein transfer versus time. (e) A sample image of serial dilutions transferred to an aminosilane slide. (TIF) [file pone.0046382.s001.tif]

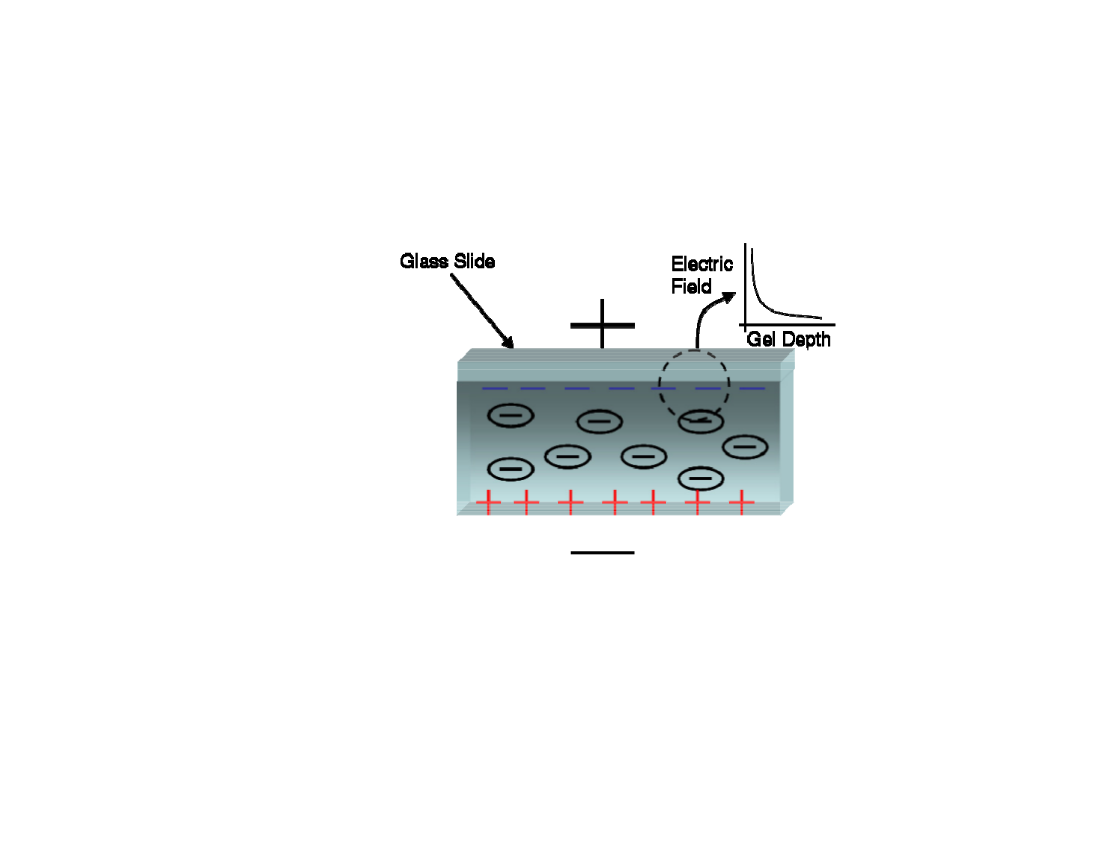

Supplement: Figure S2 — Gel transfer mechanism. (TIF) [file pone.0046382.s002.tif]
