# Supplementary material for: Protein Array Patterning by Diffusive Gel Stamping
Source: PLoS One. 2012 Oct 10;7(10):e46382. doi: 10.1371/journal.pone.0046382 (PMC3468578; doi:10.1371/journal.pone.0046382)
Supplement: Protocol S1 — Reagents, Equipment and Procedure for Diffuse Gel Stamping; Hela Cell Lysate Preparation; T47D Culture and Lysate Preparation; Western Blot Methods. Detailed figure captions. (PDF) [file pone.0046382.s003.pdf]

# Protocol for Protein Array Patterning by Diffusive Gel Stamping

Mekhail Anwar[1,2,3,6], Piyush B. Gupta[1,4], Raja Palaniapan[1,4], Paul Matsudaira[1,2,4,5,7]
[1]Whitehead Institute for Biomedical Research, Cambridge MA USA
[2]Whitehead-MIT Bioimaging Center, Cambridge MA USA
[3]Department of Electrical Engineering and Computer Science, Massachusetts Institute of Technology, Cambridge MA USA
[4]Department of Biology, Massachusetts Institute of Technology, Cambridge MA USA
[5]Department of Biological Engineering, Massachusetts Institute of Technology, Cambridge MA USA
[6]Present Address Department of Radiation Oncology, University of California, San Francisco, San Francisco CA USA
[7]Present Address Department of Biological Sciences, National University, Singapore

Reagents

1. Alexa 555 Ovalbumin (Invitrogen, Carlsbad CA)
2. Cy3 Streptavidin (Invitrogen, Carlsbad CA)
3. 10% Tris glycine gel (Invitrogen, Carlsbad CA)
4. Simply-Blue Coomassie Stain (Invitrogen, Carlsbad CA)
5. Streptavidin magnetic beads (Invitrogen, Carlsbad CA)
6. Aminosilane glass slides (Corning, Corning NY)
7. MATLAB software (Mathworks, Natick, MA)
8. biotinylated BSA, BSA (Sigma, St. Louis, MO)
9. Mini-Gel casting system, SDS mini-gel (BioRad, Hercules, CA)
10. Lifterslip (VWR, West Chester, PA)
11. anti-phosphotyrosine 635 antibody (Santa Cruz Biotechnology, Santa Cruz, CA)
12. anti-ubiquitin 488 antibody (Santa Cruz Biotechnology, Santa Cruz, CA)

Equipment

1. QSTAR Mass Spectrometer (Applied Biosystems, Foster City, CA)
2. Bioworks Browser (v3.2) (Applied Biosystems, Foster City, CA)
3. Axon 4000A Microarray Scanner (Molecular Devices, Sunnyvale CA)
4. Axon 4000B microarray scanner (Molecular Devices, Sunnyvale CA)
5. Typhoon Imager (GE Biosciences, Piscataway, NJ)

Procedure

1. Polyacrylamide gels, with the appropriate samples loaded, are run in an SDS-Page gel.

2. The gels are removed from their castings by removing only one of the casting plates, leaving the gel bounded by plastic or glass on one side, and open to the air on the other.
3. The gels are allowed to dry for between 5 and 10 minutes to eliminate excess buffer. The gels should remain moist at all times, but not have visible drops of buffer on them.
4. The gels are laid with the single sided casting down on one plate of the gel-transfer apparatus. Amino silane slides are then laid on top of the gel, with care to eliminate any air bubbles between the gel and the slide by applying subtle pressure to the slide.
5. The previously removed casting side is then placed on top of the gel, and the cover of the transfer apparatus is closed.
6. A moist chem-wipe is placed near by to ensure a humid environment so the gel does not dry out overnight.
    i. Drying of the gel can cause gel shrinkage, and this will distort the transfer image, as well as disrupt the contact surface reducing or eliminiating transfer.
7. After the appropriate amount of time, the slides are removed and washed in PBS 3 times
    i. This is accomplished by dipping them into a petri-dish containing pbs and placing the dish on a rocker for 5 minutes.
    ii. The slides are then transferred to a new dish, and the process is repeated for a total of three time.
        1. It is important that the slide does not dry, otherwise this will cause streaking.
8. The slides are then blocked by placing them into a dish containing PBS with 5% w/v BSA, on rocker table for one hour at room temperature.
    i. A pause can be taken at this step by placing the dish in the cold room overnight, and allowing the block to proceed at 4C overnight..
9. The slides are then washed as in #7.
10. The slides are dried by placing them in a slide spinner and spinning them until dry.
11. To label the slides, the appropriate dilution of antibody (typically 1:1000) is placed in 1 mg/ml BSA/PBS and mixed.
12. A lifter slip is placed over the slide and 60 ul of antibody solution is allowed to wick under the slide.
13. The combined slide/lifterslip is then placed into a petri-dish with a moist chem-wipe. The dish is then closed, and sealed with tape. The dish is covered with tin-foil.
    i. A pause can occur at this step by placing the dish in the cold room over night.
14. The antibody is allowed to incubate for 1 hour.
15. The slide is washed (#7) and dried (#10).
16. The slide is then scanned in the appropriate microarray scanner.
    i. The slide can be kept for a long period of time (> 6 months) if light shielded and stored in a vacuum desiccator.

**Supplementary Methods**

Hela Cell Lysate Preparation

Hela cell lysate was obtained by culturing Hela cells in 10% FBS / DMEM to 80% confluence in a 10 cm plate. Lysis buffer and sample buffer were made according to the protocol 9419 from Cell Signaling Technology. The plate was washed two times in ice cold PBS, and 200 µl of ice cold lysis buffer was added. Cells were scraped and placed in a 1.5 ml screw-top tube, and spun at 14k for 30 minutes at 4 degrees.

T47D Culture and Lysate Preparation

Breast cancer epithelial cells (T47D) were cultured in SILAC media (Pierce) with $^{13}C_6$ L-Lysine and $^{13}C_6$, $^{15}N_4$ L- Arginine ("heavy") and normal ("light") DMEM with 10% Dialyzed FBS. Cells were grown for five population doublings in heavy and light media to insure incorporation of heavy amino acids. Cells were grown to confluence in 15 cm plates and serum starved for 36 hours. Cells grown in light media were treated with Heregulin for 5 minutes and 3 hours. Cells grown in heavy media were used as the control, and not exposed to Heregulin.

Lysis and sample buffer was prepared as per the protocol for product 9419 (Cell Signaling Technology). Cells were then washed twice with chilled phosphate buffer saline (PBS), and 1.25 ml of lysis buffer was added. Plates were rocked on ice for five minutes, and cells were removed with a cell scraper. Cells were sonicated four times for five seconds, and spun for 30 minutes at four degrees on a table top centrifuge. The supernatant was removed and equal volumes (600 µl) of control lysate was added to 600 µl of each of the treated samples (10 minutes and 3 hours). To each 1.5 ml vial, 100 µl of anti-phosphotyrosine beads (Cell Signaling Technologies) was added. Immunoprecipitation was carried out according to the protocol 9419 from Cell Signaling Technology.

Western Blot Methods

The transfer to nitrocellulose was done under semi-dry conditions, and was transferred for 40 minutes under 80 mA of constant current. The nitrocellulose was blocked with BSA (5%) with 1% NFDM in TBS for 1 hour. The membrane was washed 3 times in 0.1% Tween/TBS and hybridized with 1:1000 Avidin HRP (Biorad) for 1 hour. The membrane was washed 3 times with 0.1% Tween/TBS and incubated with ECL Plus (GE Biosciences), and imaged with film (Kodak).

**Supplementary Figure Captions**

**Supplementary Figure 1: Characterizing Transfer Efficiency**

(a) Protein transfer versus concentration. The intensity of the transferred band is plotted on a log-log plot versus the amount of protein in the gel. The results show a linear relationship between the amount of protein loaded on the gel, and the amount transferred to the slide.
(b) Protein transfer versus electric field. To test the effect of the electric field on transfer, the same experiment as in (a) is repeated, but with an identical set of gels transferred with 50 volts across the gel. The results show a linear dependence on protein concentration, but no dependence on electric field. This is consistent with the theory that the electric field is screened in the gel, and the method of transfer is diffusion based.
(c) Protein transfer versus gel thickness. The intensity of transferred bands is plotted versus the amount of protein in the gel for varying gel thicknesses (0.5 mm, 0.75 mm, 1.0 mm, 1.5 mm). The thinner gels resulted in a greater transfer efficiency. This is consistent with the diffusive model, as the concentration of protein will be increased in a thinner gel.
(d) Protein transfer versus time. The transfer intensity for various concentrations is plotted as a function of time. The data is fit to a first order exponential, indicating that the transfer follows simple diffusive kinetics, eventually reaching equilibrium with the slide.
(e) A sample image of serial dilutions transferred to an aminosilane slide.

**Supplementary Figure 2: Gel transfer mechanism.**

A cellular lysate is separated on a standard SDS-PAGE gel, which is then placed on a functionalized glass slide. The proteins are allowed to diffuse onto the slide. An applied electric field induces the ions to form a charge screen, resulting the electric field only penetrating only on the order of the Debye length, (typically less than 1 nm), resulting in proteins traveling solely by diffusion.
