# Supplementary material for: Protein Array Patterning by Diffusive Gel Stamping
Source: PLoS One. 2012 Oct 10;7(10):e46382. doi: 10.1371/journal.pone.0046382 (PMC3468578; doi:10.1371/journal.pone.0046382)
Supplement: Data S1 — Table 1: Proteins from Extracted Gel Band. Table 2: Table 2: Proteins from immunoprecipitated Gel band. (PDF) [file pone.0046382.s004.pdf]

Supplementary Data

Protein Description	MOWSE Score	Protein Mass	Protein Coverage
(P11142) Heat shock cognate 71 kD protein (Heat shock 70 kD protein 8)	448	71082	34.8
(Q59EJ3) Heat shock 70kDa protein 1A variant (Fragment)	328	78018	29.5
(Q8N1C8) Heat shock 70kD protein 9B (Mortalin-2) (Fragment)	287	74093	17.9
(P29401) Transketolase (EC 2.2.1.1) (TK)	229	68519	28.3
(P17066) Heat shock 70 kD protein 6 (Heat shock 70 kD protein B~)	224	71440	13.8
(P02545) Lamin-A/C (70 kD lamin) (NY-REN-32 antigen)	203	74380	31.5
(Q6FG89) G22P1 protein	202	70114	24
(P34931) Heat shock 70 kD protein 1L (Heat shock 70 kD protein 1-like) (Heat shock 70 kD protein 1-Hom) (HSP70-Hom)	201	70730	19
(P26038) Moesin (Membrane-organizing extension spike protein)	170	67761	24.5
(P11940) Polyadenylate-binding protein 1 (Poly(A)-binding protein 1) (PABP 1)	147	70854	20.4
(Q2KHP4) Hypothetical protein	141	72492	16.6
(P20700) Lamin-B1	114	66522	20.7
(P04843) Dolichyl-diphosphooligosaccharide--protein glycosyltransferase 67 kD subunit precursor (EC 2.4.1.119) (Ribophorin I) (RPN-I)	112	68641	15.2
(P08133) Annexin A6 (Annexin VI) (Lipocortin	99	76037	24.6

VI) (P68) (P70) (Protein III) (Chromobindin-20) (67 kD calelectrin) (Calphobindin-II) (CPB-II)			
(O60506) Heterogeneous nuclear ribonucleoprotein Q (hnRNP Q) (hnRNP-Q) (Synaptotagmin-binding, cytoplasmic RNA-interacting protein) (Glycine- and tyrosine-rich RNA-binding protein) (GRY-RBP) (NS1-associated protein 1)	98	69788	14.3
(Q12931) Heat shock protein 75 kD, mitochondrial precursor (HSP 75) (Tumor necrosis factor type 1 receptor-associated protein) (TRAP-1) (TNFR-associated protein 1)	93	80345	14.2
(Q9BV64) HNRPR protein (Heterogeneous nuclear ribonucleoprotein R)	90	71456	11.3
(Q6NUR7) Villin 2 (Ezrin)	83	69313	13.1
(P35241) Radixin	83	68635	8.7
(Q15582) Transforming growth factor-beta-induced protein ig-h3 precursor (Beta ig-h3) (Kerato-epithelin) (RGD-containing collagen-associated protein) (RGD-CAP)	81	75261	3.7
(Q9UJU1) Cytovillin 2 (Fragment)	73	16294	22
(Q96AE4) Far upstream element-binding protein 1 (FUSE-binding protein 1) (FBP) (DNA helicase V) (HDH V)	71	67602	10.1
(P13667) Protein disulfide-isomerase A4 precursor (EC 5.3.4.1) (Protein ERp-72) (ERp72)	70	73229	6.7
(Q5D0D7) ALB protein	69	73881	6.9
(Q8IW48) SDHA protein	56	57283	11
(P52272) Heterogeneous nuclear ribonucleoprotein M (hnRNP M)	55	77618	5.2

(Q0JS26) Hypothetical protein PDLIM5 (Fragment)	50	54370	1.8
(Q96I24) Far upstream element-binding protein 3 (FUSE-binding protein 3)	49	61944	1.4
(P03951) Coagulation factor XI precursor (EC 3.4.21.27) (Plasma thromboplastin antecedent) (PTA) (FXI) [Contains: Coagulation factor XIa heavy chain; Coagulation factor XIa light chain]	48	72116	1.9
(Q03252) Lamin-B2	48	67762	2.7
(Q9NTK6) Hypothetical protein DKFZp761K0511	46	85189	4.5
(Q14683) Structural maintenance of chromosome 1-like 1 protein (SMC1alpha protein) (Sb1.8)	45	143771	3.8
(P29966) Myristoylated alanine-rich C-kinase substrate (MARCKS) (Protein kinase C substrate, 80 kD protein, light chain) (PKCSL) (80K-L protein)	43	31576	5.7
(P17844) Probable ATP-dependent RNA helicase DDX5 (EC 3.6.1.-) (DEAD box protein 5) (RNA helicase p68)	42	69618	10.1
(Q59F66) DEAD box polypeptide 17 isoform p82 variant (Fragment)	42	81701	4.3
(Q16630) Cleavage and polyadenylation specificity factor 6 (Cleavage and polyadenylation specificity factor 68 kD subunit) (CPSF 68 kD subunit) (Pre-mRNA cleavage factor Im 68 kD subunit) (Protein HPBRII-4/7)	40	59344	2.5

Table 1: Proteins from Extracted Gel Band

Protein Description	MOWSE Score	Protein Mass	Protein Coverage
(Q8IUK7) ALB protein	60	46442	3.8

Table 2: Proteins from immunoprecipitated Gel band.
